# A Rare Case of Low-grade Appendiceal Mucinous Neoplasm: A Case Report

**DOI:** 10.7759/cureus.3980

**Published:** 2019-01-29

**Authors:** Hector H Gonzalez, Kimberly Herard, Maria C Mijares

**Affiliations:** 1 Internal Medicine, Florida Atlantic University Charles E. Schmidt College of Medicine, Boca Raton, USA

**Keywords:** pseudomyxoma peritonei, appendix, mucin, low-grade appendiceal mucinous neoplasm

## Abstract

Low-grade appendiceal mucinous neoplasm (LAMN) is a rare malignancy with symptoms varying depending on the clinical manifestations. The most worrisome complication of this particular neoplasm is seeding of mucin into the adjacent peritoneum leading to pseudomyxoma peritonei (PMP). There is a lack of standardized treatment approach; however, an appendectomy-only approach is currently being used for the resection of non-metastatic disease. We present an unusual case of a 67-year-old male found to have LAMN status post elective appendectomy, six months after being treated for an appendiceal abscess.

## Introduction

Low-grade appendiceal mucinous neoplasm (LAMN) is a rare malignancy accounting for 1% of gastrointestinal neoplasms and is found in less than 0.3% of appendectomy specimens [1-2]. LAMNs are diverse and can be classified as colonic-type, mucinous adenocarcinoma, goblet cell adenocarcinoma, or neuroendocrine carcinoma [3]. Mucinous adenocarcinoma accounts for the majority of cases according to the literature [2]. This malignancy is commonly an incidental finding during operative exploration and is often diagnosed late.

Gross examination of LAMN may be unremarkable or may appear as a mucin-filled, cystically dilated tissue. The appendix wall may appear thin, fibrotic, hyalinized, or calcified with a smooth or granular appearance [4]. Similar to polyps found in the colon, LAMN can be classified as villous or flat with atrophied lymphoid tissue. Neoplastic tissue growth occurs in a “pushing” invasion pattern wherein no tumor budding or single-cell invasion is noted [4]. In 2010, the World Health Organization (WHO) improved the cytoarchitectural classification in hopes of accurate diagnosis and appropriate treatment modalities [1].

LAMNs are associated with diverticula, herniations, dissections, and rupture [4]. The most feared complication is seeding of mucin into the adjacent peritoneum, leading to pseudomyxoma peritonei (PMP), associated with a high rate of mortality [1-2]. Seeding into the peritoneum occurs in the late stages of the disease. Our case was unique due to the findings of periappendiceal acellular mucinous deposits without evidence of perforation. 

## Case presentation

**Figure 1:**
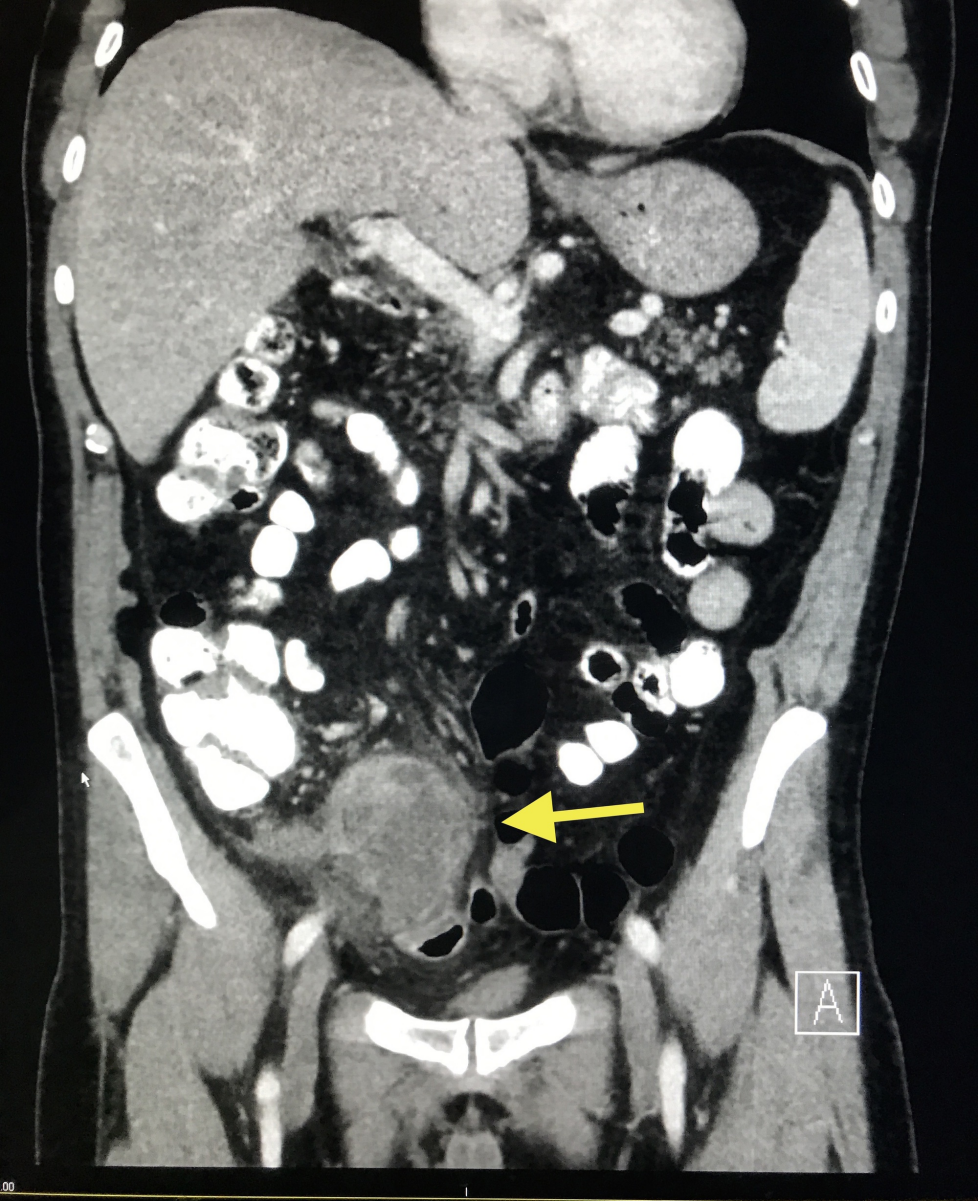
CT scan of the abdomen/pelvis showing perforated acute appendicitis with a 7-cm abscess and large phlegmonous change (yellow arrow) in the right lower quadrant with secondary inflammatory changes CT: computed tomography

A 67-year-old Caucasian male with a past medical history of gastroesophageal reflux disease presented to the hospital for an elective appendectomy. Of note, he was admitted six months prior for acute appendicitis with perforation and abscess formation. At that time, computerized tomography (CT) scan of the abdomen/pelvis revealed a perforated appendix with a 7-cm abscess in the right lower quadrant (Figure 1).

**Figure 2:**
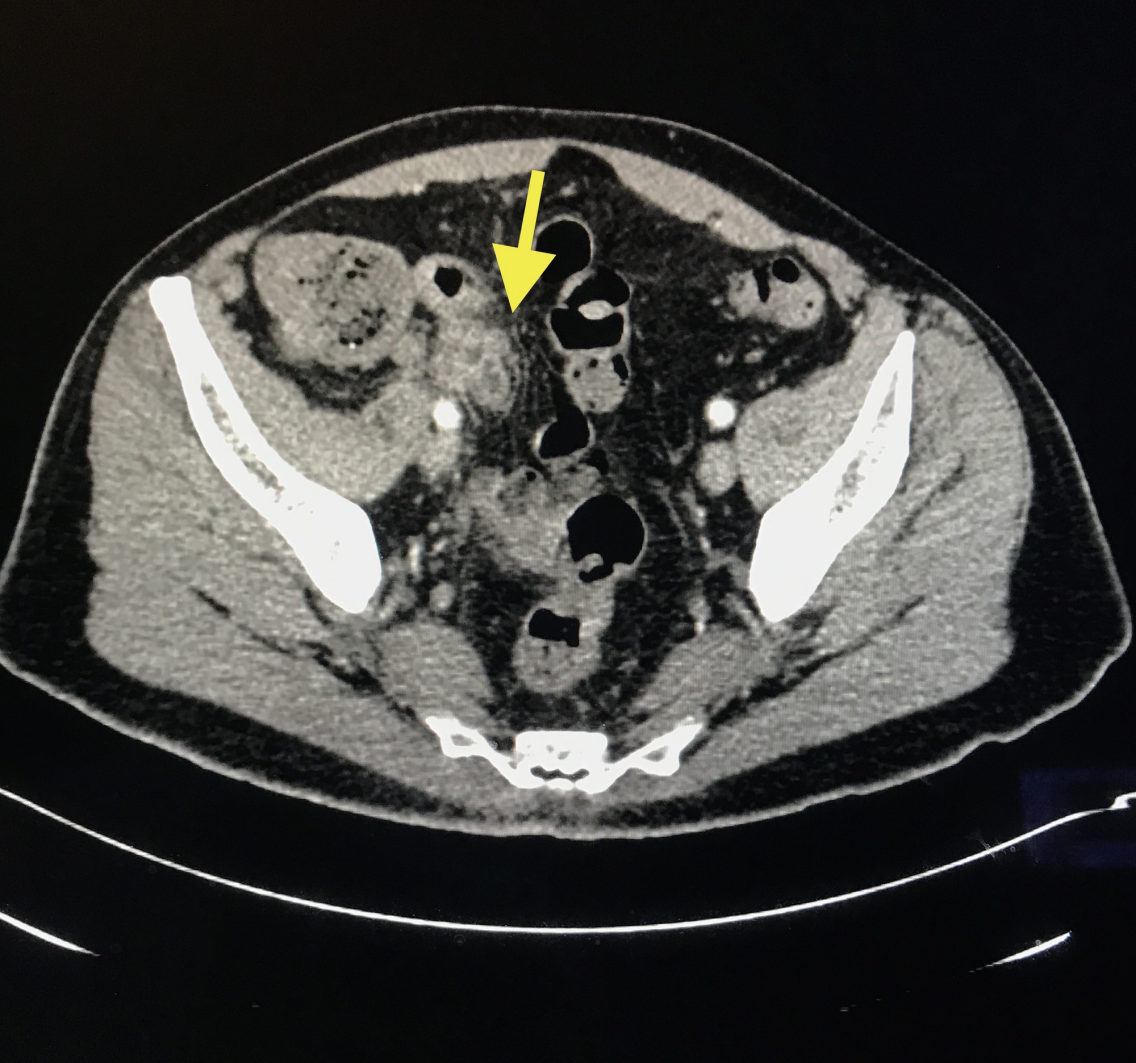
CT scan of the abdomen/pelvis showing mass measuring 3 cm at the tip of the appendix (yellow arrow), with the absence of collection Findings suspicious for post-inflammatory changes within the area; however, malignancy cannot be excluded. CT: computed tomography

The patient was medically managed with 10 days of ertapenem and percutaneous drainage. Follow-up CT scan of the abdomen/pelvis eight weeks post-drainage showed an intra-appendiceal mass, representing chronic inflammatory changes versus tumor (Figure 2).

**Figure 3:**
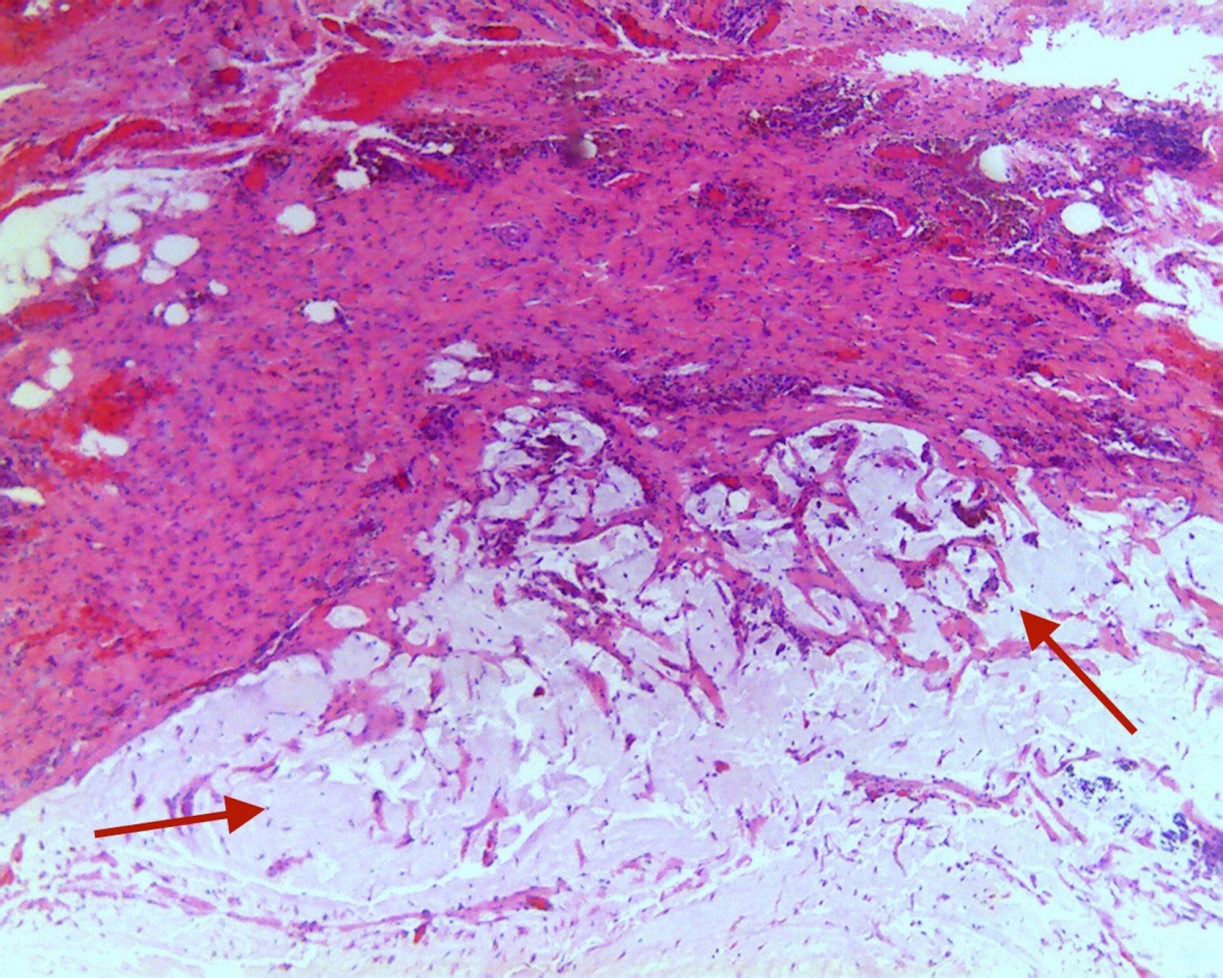
Hematoxylin and eosin stain showing diffuse necrosis with invasive mucinous adenocarcinoma exhibiting extensive mural replacement by large, irregular, dissecting pools of mucin (red arrows), containing free-floating neoplastic epithelium

The patient underwent evaluation of possible underlying tumor with complete blood count, basic metabolic panel, and carcinoembryonic antigen (CEA) which were all unremarkable. Colonoscopy was performed, which did not show any abnormality at the appendiceal orifice. Unfortunately, the patient was lost to follow-up prior to his elective appendectomy despite recommendations for surgery at that time. The patient was reevaluated after lost to follow-up and decided to undergo laparoscopic appendectomy. Laparoscopic appendectomy revealed an intact appendix with a visualized bulbous tip and no evidence of metastatic disease. Gross examination of the specimen revealed a vermiform appendix measuring 6.5 x 1.3 cm. Sectioning of the specimen showed a 1.3 x 0.6-cm mucinous area surrounding the distal aspect of the appendix. Specimen pathology revealed LAMN with rare diverticula into the appendiceal wall and an extensive 1.3-cm area of periappendiceal acellular mucinous deposits. Extensive mucinous pools were identified in the periappendiceal tissue without evidence of perforation (Figure 3). Mucinous epithelium was absent in the mucin pools. 

The patient was stable postoperatively with no surgical complications. Outpatient follow-up was recommended with a CT scan of the abdomen and pelvis in six months.

## Discussion

LAMNs are rare adenomas localized in the appendix or the surrounding appendiceal mucosa wall. These neoplasms are more commonly diagnosed in men, particularly in the sixth decade of life. Patients with LAMN can present with abdominal pain, intussusception, and obstruction. However, LAMNs are often incidentally found in asymptomatic patients. Complications of LAMN include intussusception, ureteral obstruction, volvulus, small bowel obstruction (SBO), rupture, and PMP [1-2].

Often, this malignancy is misdiagnosed as acute appendicitis, retroperitoneal tumors in the right iliac fossa, or an adnexal mass [2]. Imaging modalities for diagnosis include ultrasound (US) and CT, with CT as the most commonly used radiographic interpretation for preoperative diagnosis. The common abdominal CT findings include cystic dilation within the appendiceal lumen with wall calcifications and irregular appendiceal wall thickening as demonstrated in our case. Grossly, specimens of LAMN include hyalinization and fibrosis of the appendiceal wall with a grossly swollen appendix secondary to mucinous accumulation [1-2,4]. LAMNs less than two centimeters (cm) are rarely malignant and are classified as benign simple or retention mucoceles. Masses larger than 6 cm present with a higher risk of malignant cells, a higher risk of appendiceal perforation, and development of PMP [2]. Histological evidence of LAMN includes atypical glandular cells and epithelial cells with “pushing invasion” of malignant cells creeping into the appendiceal wall with possible diverticular formation [4]. Mucinous, colonic, and goblet cells are also often identified within LAMN [5]. Elevated CEA, Ca 19-9, and Ca-125 may be detected in 56.1-67.1% of patients with LAMN [6]. These tumor markers can also be used for the surveillance of peritoneal malignancy following surgical or medical intervention. There is also a 35% risk of a concurrent GI malignancy in patients with LAMN [5]. 

Controversy remains on the best surgical approach (laparoscopic vs open), adjuvant therapy, follow-up duration, and imaging technique. The goal of management of LAMN includes the prevention of rupture, seeding, and development of PMP [2]. The practice of right hemicolectomy in the absence of lymph node metastasis has been replaced, with an appendectomy only approach used for the treatment of benign appendiceal mucoceles. Upon discovery of infiltration of malignancy into submucosa or with the presence of lymph node metastasis, right hemicolectomy with or without omentectomy may be performed [3]. In our case, there was no pathological evidence of malignancy infiltration into the bowel submucosa or lymph node metastasis and no evidence of malignant cells in the mucin pools in the periappendiceal tissue. Thus, further surgical and adjuvant therapies were not required in our patient. Our patient underwent a laparoscopic procedure that allows magnification of the surgical field and rapid patient recovery. The risk of peritoneal seeding increases with the removal of specimens through the port site but can decrease the risk of seeding overall as reported by Fujuni et al. [7]. Lymph node metastasis is a rare occurrence in only 4.2% of patients but would require an aggressive treatment [8].

PMP is a complication of mucinous LAMN that can develop from peritoneal seeding in 20% of patients with a mucinous adenoma. It can be diagnosed using various modalities such as ultrasonography, CT scan, and magnetic resonance imaging depicting the presence of gelatinous mucinous nodules in the peritoneal cavity [1]. However, these imaging modalities have only been shown to identify up to 29% of adenomas prior to surgical intervention [6]. Histopathology of PMP depicts epithelial cells and mucin in the peritoneum [4]. Further advances in biomarkers and molecular genetics demonstrate CDX2, MUC-2, CK 20, β- catenin, CEA, CA 19-9, and KRAS mutations identified in hopes of improving early identification [1]. The five-year survival rate for PMP is 25% [9]. Aggressive treatments are required for PMP including appendectomy, as the appendix is the source of malignant cells in 95% of cases [1]. Aggressive strategies also include cytoreductive surgery and hyperthermic intraperitoneal chemotherapy [6].

Surveillance of patients with LAMN incorporates radiographic imaging every six months post appendectomy for two years for adequate monitoring of tumor recurrence and complications associated with PMP [10]. Accurate pathological assessment and classification of LAMN are important to assess for malignancy risk, seeding, recurrence, and patient prognosis [1]. For patients with a high risk of disease progression, follow-up should continue for the first five years after diagnosis of LAMN. High-risk patients include those with evidence of infiltration of malignancy into submucosa or with the presence of lymph node metastasis. Additional surveillance and treatment studies are needed, but until then, the treatment for LAMNs will remain inconsistent due to a lack of standardized interventions based on diagnostic criteria. Close follow-up was recommended for our patient, due to increased risk of LAMN with acellular mucin deposits outside appendix developing recurrence or PMP. Follow-up should continue for five to 10 years with physical exams, annual CT, and monitoring of tumor markers. The five-year survival rate for localized LAMN is 95%.

## Conclusions

Overall, further studies are needed for a more definitive method of diagnosis, treatment, and monitoring of LAMN. Diagnosis to date varies by imaging modality, the tumor markers utilized, and classification of disease. There remains a lack of standardization for post-treatment surveillance lengths and methods. This case presents the importance of developing a high index of suspicion regarding the development of appendiceal malignancies and choosing the appropriate surgical or medical treatment modality to prevent recurrence, seeding, and later development of PMP.
